# Injury patterns in patients with severe traumatic brain injuries from motor crashes admitted to Mulago hospital accidents & emergency unit

**DOI:** 10.1016/j.afjem.2023.03.003

**Published:** 2023-04-23

**Authors:** Joseph Kalanzi, Lee Wallis, Mary Nabukenya, Erasmus Okello, Doreen Okong, Stella Namirembe

**Affiliations:** a Department of Anaesthesia, Critical care and Emergency Medicine, College of Health Sciences, Makerere University, Kampala, Uganda; b Division of Emergency Medicine, University of Cape Town, Cape Town South Africa

**Keywords:** Road traffic crash, Brain injuries, Polytrauma

## Abstract

**Background:**

The global burden of Road Traffic Crashes (RTC) is increasing. Uganda has one of the highest rates of RTCs in Sub-Sahara. Victims of RTCs sustain varying degrees of injuries depending on factors including the velocity at time of impact, protective gear; and if it was a motorcycle–motorcycle or motorcycle–vehicle crash. High speed collisions can result in severe forms of injuries and polytrauma. Some injuries are undetected.

**Methods:**

A cross sectional study was carried at Mulago Hospital Accidents & Emergency Unit, between November 2021 and February 2022; on all adult patients (≥18 years) with severe head injury from motor road traffic crashes. The study looked at injury patterns and assessed the relationship of polytrauma in patients with severe head injury to the mechanism of injury (motorcycles versus vehicles). Data were extracted from patient charts using a validated data abstraction tool and complete head to toe physical examination was carried out and injuries recorded. Data were analysed to determine the relationship of polytrauma in patients with severe head injury to the mechanism of injury.

**Results:**

The participants were predominantly males with a population median age of 32 (25-39). The commonest modes of transportation of patients to the hospital were Police Pickup trucks (40%) and ambulance (36.1%). Among motorcycle RTCs, (19.2%) wore helmets; 21.2% had protective gear; with injury identified mainly in; the limbs (84.8%), neck (76.8%), chest (39.4%), and abdomen (26.3%). Patients from vehicle RTCs were 19% more likely to have polytrauma compared to patients from motorcycle RTCs.

**Conclusions:**

This study showed that patients who sustain severe traumatic brain injuries from vehicle crashes have an increased likelihood of having multiple injuries, compared to patients from motorcycle RTCs. For motorcycle users, injuries mostly affect the limbs. At particular risk are motorcyclists who do not wear helmets and protective coveralls.

## African Relevance

• Disproportionately high burden of injuries and deaths from road traffic crashes in Africa.
• Accidents and Emergency units manage large volumes of patients with severe forms of injury, with limited data describing the injury patterns.
• Emergency care systems underdeveloped with limited guidelines on trauma care.
• Need for data to support implantation of public health prevention initiatives towards motorcycle riders and users wearing protective gear.

## Introduction

The burden of injuries and deaths resulting from Road Traffic Crashes (RTC) is extremely high. It has a global distribution, but its incidence and impact are greatest in low- and middle-income countries [1]. Victims of RTC are a common presentation to Accident and Emergency (A&E) Units. The Global Status Report on road safety 2015 [2] approximated that the annual road traffic deaths have reached 1.35 million, disproportionately affecting pedestrians and motorcyclists, especially those in developing countries. Victims sustain injuries to varying degrees and extents with some sustaining multiple injuries or polytrauma. Polytrauma is a short verbal equivalent used for severely injured patients usually with associated injury such as two or more severe injuries in at least two areas of the body [3]. These include injuries to the head, spine, chest, abdomen, pelvis, and limbs. Studies have attempted to show the association between various injuries sustained from motorcycle crashes [4]. In Uganda, 41% of the RTC involve motorcycles [5]. The two-wheeled motorbike transport has become an increasingly popular mode of transport in the country. A 2004 study conducted at Mulago in the surgical and orthopaedic wards reported that a proportion (20%) of the victims of road traffic crashes involving motorcycles had poly-trauma [6].

Several studies have been conducted in Uganda involving victims of motorcycle road traffic crashes identifying various injuries commonly such as maxillofacial injuries [7] and orthopaedic injuries [6]. A 2019 study looking at motorcycle injuries in Uganda reported a prevalence of 71.1% of head injuries [8]. Outcomes among these patients have been reported to include neurological impairment, moderate disability (13.5%), severe disability (2.1%) and death (10.8%) [8]. Due to the lack of protective gear, there are cases of severe forms of injuries sustained. These injuries contribute significantly to the mortality of otherwise able-bodied people as well as long term morbidity for the survivors. Victims can sustain multiple injuries (polytrauma) requiring attention from specialists from different disciplines initially during the A&E unit stay and during admission on the specialist wards. Some injuries are missed during initial assessment and care with attention given to the outstanding severe injuries. Patients with severe head injuries, Glasgow Coma Scale < 8, are at higher risk of missed injuries [9].

Motorcyclists and pedestrians are a particular vulnerable group at risk of injuries. The 2018 WHO report on the global incidence of traumatic brain injury, documented that approximately sixty-nine million individuals globally are estimated to sustain a head injury each year [10]. Patients sustain varying degrees of injuries with some cases involving polytrauma/ multiple injuries. A 2008 study carried out in Turkey documented frequencies in motorcycle related injuries as musculoskeletal system (50%), skull (48.6%), maxillofacial (17.9%), thoracic (7.1%), vertebral (4.7%), and abdominal injuries (2.8%) [11].The proportion of head injuries resulting from these crashes is disproportionately higher (56%) in Africa (Dewan et al., 2018). Reports from Kenya indicate fatalities of 2893 in 2017, with 17% being motorcyclists [12]. Uganda Police data reported that between 2012 and 2014, there were 53,147 road traffic injuries and 8,906 fatalities [13]. The introduction of motorcycles as a popular means of transport contributed to the already high incidence of RTC. Trauma resulting from RTC can be isolated singular injuries or to multiple sites (polytrauma). Motorcycle crashes have been associated with polytrauma patterns of injury [6]. Studies have been conducted at Mulago Hospital to describe the injury patterns among motorcyclists, looking at injuries to the chest, abdomen, spine, and extremities [14]. A 2020 study on head injuries documented a high proportion of 56.8% of cases of severe head injuries seen at Mulago Hospital to be a result of RTCs [15]. Mild injuries to the limbs also contribute significantly (61.6%), alongside neck and head injuries (42.0%) in patients seen at Mulago Hospital [16].A 2020 study at Mulago Hospital documented that many patients with severe head injuries die while in the emergency unit, due to severity of head injuries alone or multiple injuries that compound the head injury [15]. Orthopaedic injuries specifically fractures are also often sustained.69 million individuals worldwide are estimated to sustain a traumatic head injury each year, with the proportion resulting from RTCs greatest in Africa and Southeast Asia [10]. The outcomes of death or morbidity can be averted if clinicians were aware of the patterns of injuries that should be suspected in cases of severe head injuries. Despite Advanced Trauma Life Support guidelines on the approach to patients with trauma, some injuries in patients with severe head injuries, could be missed during the assessment and care in the A&E unit since attention is drawn to the head injury. These injuries could be responsible for deaths or long-term morbidity and physical impairment if not detected early. The exact pattern of polytrauma in severe head injured patients post motorized crashes is unknown.

### General objective

To describe injury patterns in patients with severe traumatic brain injuries from Road Traffic Crashes admitted to Mulago Hospital Accidents & Emergency unit.

### Specific Objectives

1 To describe the injury patterns of motorcycle and motor vehicle crash patients admitted at Mulago Hospital Accidents & Emergency Unit.
2 To compare the prevalence of polytrauma in patients with severe traumatic brain injuries sustained from motorcycle crashes against those sustained from motor vehicle crashes.

## Methods

### Study design

This was a cross sectional study design. Participants were recruited between November 2021 and February 2022.

### Study setting

The study was conducted at Mulago Hospital. The hospital is located on Mulago Hill, Kawempe division, Kampala, Uganda. The hospital is a multi-speciality facility that serves as the National Referral Hospital for Uganda with a population of forty million, according to the Uganda National Population and Housing Census, 2014. The hospital has a bed capacity of 1,790 beds, although it often houses over a population of 3,000. The A&E department receives about 61,568 emergencies annually and 150 patients with severe head injuries monthly. The department also receives about five patients with polytrauma per day. The A&E has nine beds, an operating theatre for severe trauma surgeries, linked with the Intensive Care Unit for admission of patients requiring critical care: a resuscitation room with two ventilators and one transport ventilator for patient transport.

### Study population

All adult patients (≥18 years) with severe traumatic brain injury sustained from motorcycle and motor vehicle crashes admitted at Mulago Hospital A&E unit during the study period.

### Eligibility criteria

Severe Traumatic Brain Injury was defined as a Glasgow Coma Scale (GCS) score of 8 or less following nonsurgical resuscitation.

### Inclusion

All adult patients (≥18 years) with severe traumatic brain injury sustained from motorcycle and motor vehicle crashes admitted at Mulago Hospital A&E unit**.**

### Exclusion

Patients admitted in the A&E with a diagnosis of severe traumatic brain injury who were pedestrians were excluded from the study.

### Sample size estimation

The sample size that described the patterns of injuries associated with severe head injury following a motorcycle and motor vehicle crash was estimated at 150 using the formula proposed by Leslie Kish (1964) [17]

$$N=\frac{\;{\left(Z_{1-\;\frac{\alpha }{2}}\right)}^{2}*\;p\;\left(1-p\right)}{d^{2}}$$

Minimum sample size required to compare the prevalence of polytrauma in patients with severe traumatic brain injuries sustained from motorcycle crashes against those sustained from motor vehicle crashes was estimated at 160 using the formula for that compared two proportions based on proportions of multi-system trauma reported by Landes et al [18]

$$n=\frac{{\left[Z_{\alpha /2}\sqrt{2p\;\left(1-p\right))}+Z_{\beta }\sqrt{p_{1}\;\left(1-p_{1}\right)+p_{2}\;\left(1-p_{2}\right)}\right]}^{2}}{\;{\left(p_{1}-p_{2}\right)}^{2}}$$

### Sampling procedure

Consecutive sampling was used for every subject meeting the inclusion criteria until the required sample size was achieved.

### Study variables

#### Independent variables

The main predictor was severe traumatic brain injury from motorcycle crashes or motor vehicle crashes. Other independent variables in this study were age, sex, Glasgow Coma Scale, history using protective gear (helmets and coveralls) at the time of motor crash.

#### Dependent variables

The dependent variable was the counts of injuries per patient involved in motor crashes.

### Data collection

Data was collected from patient's charts in the A&E Unit Mulago National Referral Hospital using a data abstraction tool. The patients were entered into the study at 24 hours from the time of admission. Trained research assistants selected all charts of patients with a documented diagnosis of severe head injury following motorcycle and motor-vehicle crashes. They filled in the clinical findings of injuries documented in the patients’ charts by the attending doctors and conducted a full head to toe physical examination to record any other injuries. The protocol for systematic complete physical examination that was used was:

1 Patients were carefully exposed.
2 Head-to- toe examination in the following order
  ■ Head and neck
  ■ Chest
  ■ Abdomen
  ■ Pelvis
  ■ Extremities (Upper limb and lower limbs)
  ■ Spine

### Data management

Data was coded and entered using Epidata (version 3.1). Data was checked for completeness, reliability, and validity. Double data entry was used to avoid errors in the data. The principal investigator supervised and fully participated in the data collection, entry, analysis, and reporting.

### Data analysis

Data were examined in detail using univariate, bivariate and multivariate analysis in Stata (version 14, 2012) software.

#### Univariable analysis

Variables were described using frequencies and percentages for categorical variables while numerical variables were described as means with standard deviations for normally distributed continuous data or medians with interquartile ranges for continuous but skewed data.

#### Bivariable analysis

Poisson regression was used to determine the relationship of multiple injuries in patients with severe head injury to the mechanism of injury.

#### Multivariable analysis

Poisson regression was used to determine the relationship of polytrauma in patients with severe head injury to the mechanism of injury (RTCs involving motorcycles and vehicles) while controlling for several independent variables.

### Quality control

Data was validated by the principal investigator before double entry to eliminate any errors. The data collection tool was pre-tested on 5% of the sample size, from an external population.

### Ethical considerations

Written informed consent was obtained from attendants of patients with severe head injury to obtain their consent. The team protected the privacy of the patients by using unique identifiers. Ethics approval was obtained from Makerere University School of Medicine Research and Ethics Committee.

## Results

### Study profile

**Fig. 1:**
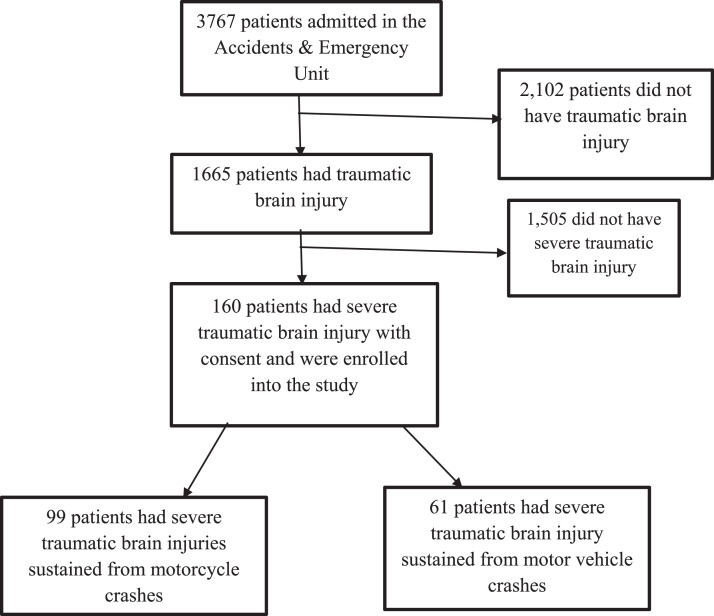
Study profile.

From November 2021 to February 2022, 3767 patients were admitted in the Accidents and Emergency Unit of Mulago National Referral Hospital. Out of this population, 1665 patients had traumatic brain injury. Fig. 1 summarises the enrolment:

### Baseline characteristics of patients with severe head injury from crashes involving motorcycles and vehicles

The median age (IQR) was 32 ([25]-39) years (Table 1). Most (> 50%) of the patients were within 30- 40 years of age. Of the 160 patients recruited; 117 (73.1%) were males. Overall, the most used modes of transportation of patients to the hospital were police pickup trucks (40.0%), ambulances (36.9%), and motorcycles (6.3%).

**Table 1:** Baseline characteristics of patients with severe traumatic brain injuries from motorcycle and vehicle crashes.

Characteristic		Motorcycle (n=99)	Vehicle (n=61)	Total (n=160)
Sex	Male	80 (80.8)	37 (60.7)	117 (73.1)
	Female	19 (19.2)	24 (39.3)	43 (26.9)
Age (years)	Median (IQR)	32 (26-40)	30 (23-38)	32 (25-39)
	18-35	63 (63.6)	42 (68.9)	105 (65.6)
	>35	36 (36.4)	19 (31.1)	55 (34.4)
Mode of transport to hospital	Ambulance	38 (38.4)	21 (34.4)	59 (36.9)
	Private vehicle	14 (14.1)	13 (21.3)	27 (16.9)
	Police pick-up	41 (41.4)	23 (37.7)	64 (40.0)
	Motorcycle	6 (6.1)	4 (6.6)	10 (6.3)
Protective Helmet		19 (19.2)	N/A)	N/A
Protective coverall		21 (21.2)	N/A)	N/A
Glasgow Coma Scale	3-5	21 (21.2)	11 (18.0)	32 (20.0)
	6-8	78 (78.8)	50 (82.0)	128 (80.0)

Injury patterns of motorcycle users admitted at Mulago Hospital Accidents & Emergency Unit.

### Use of protective gear

**Fig. 2:**
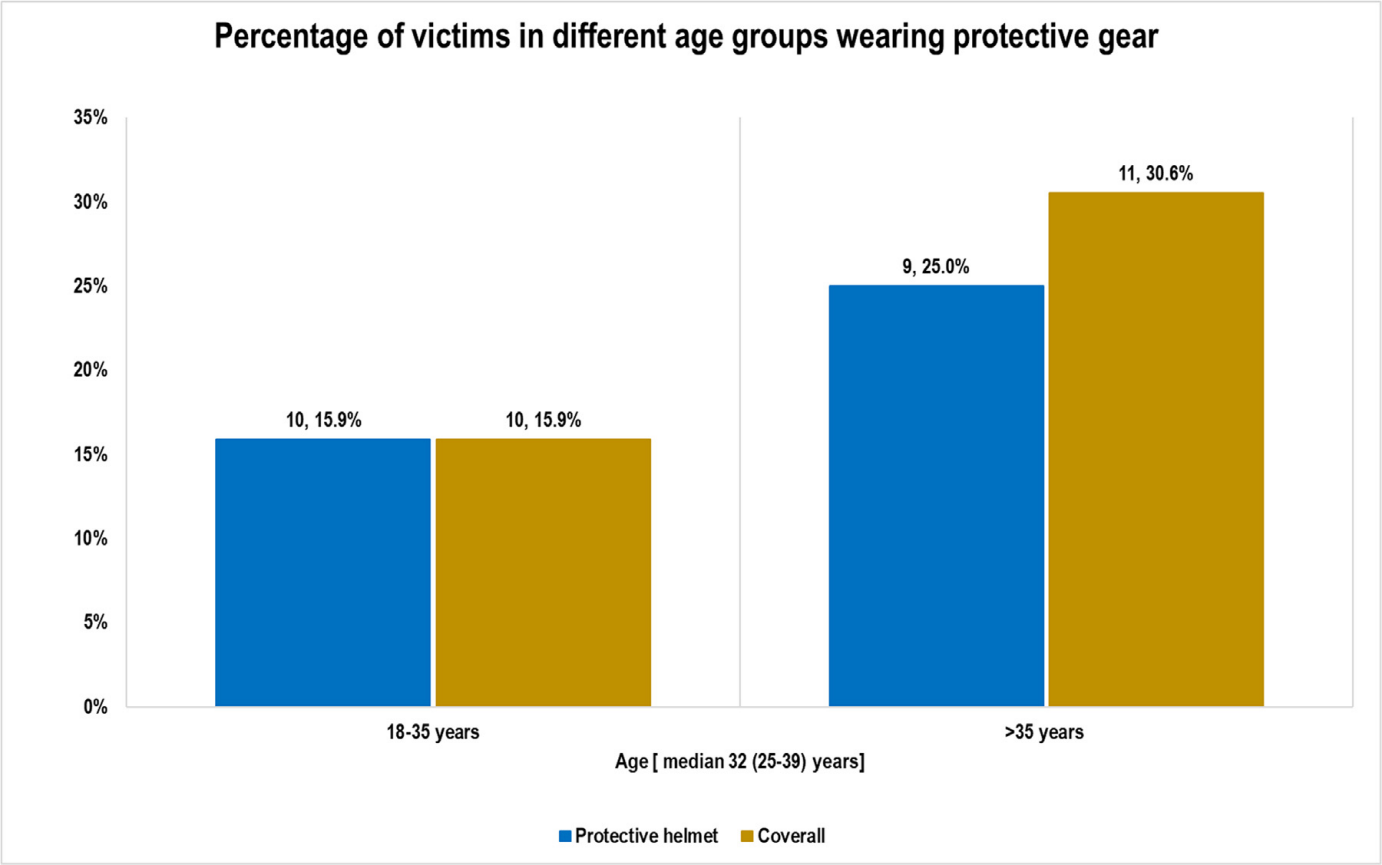
Bar chart showing percentage of victims in different age groups with severe TBI from motorcycle crashes who were wearing protective gear.

Among motorcycle crash patients, helmet use was reported in only 19.2% of cases; and only 21.2% wore protective gear (jacket and or coveralls). In both age groups, use of protective helmet was exceptionally low; 15.9% (18-35years) compared with 25% (>35 years) (Fig. 2). Use of protective coverall was also low; 15.9% (18-35years) compared to 30.6% (>35 years).

### Distribution of injuries found in patients with severe traumatic brain injuries from motorcycle crashes

In patients from motorcycle RTCs, the body regions that had the most associated injuries were the limbs (84.8%); neck (76.8%), chest (39.4%) and abdomen (26.3%). (Table 2). The least affected regions were the spine and pelvis at 6.1%.

**Table 2:** Table showing distribution of injuries by location in patients with severe traumatic brain injuries from motorcycle crashes.

Characteristic *Body site*	Motorcycles (n=99)	Frequency (%)
Neck	76	76.8%
Chest	39	39.4%
Abdomen	26	26.3%
Pelvis	6	6.1%
Limbs	84	84.8%
Spine	6	6.1%

### Count of injuries associated with severe traumatic brain injuries sustained from motorcycle crashes

Overall, soft tissue injuries accounted for 67.4% and skeletal injuries accounted for 22.3%. The neck region was frequently injured (35.0%); mostly with soft tissue injuries (31.1%). These included hematomas, bruises, and lacerations. Most skeletal injuries (fractures and dislocations) were reported in the lower limbs (19.9%), compared to 13.8% in the upper limbs. The most common injuries identified in the chest region were majorly soft tissue injuries (14.2%). The rest included skeletal injuries [fractures] (2.8%); haemothorax (0.8%) and pneumothorax (0.4%). The spine had the least associated injuries (1.9%); of which 1.5% were skeletal (vertebral fractures/ dislocations).

Prevalence of polytrauma in patients with severe traumatic brain injuries sustained from motorcycle versus motor vehicle crashes.

**Fig. 3:**
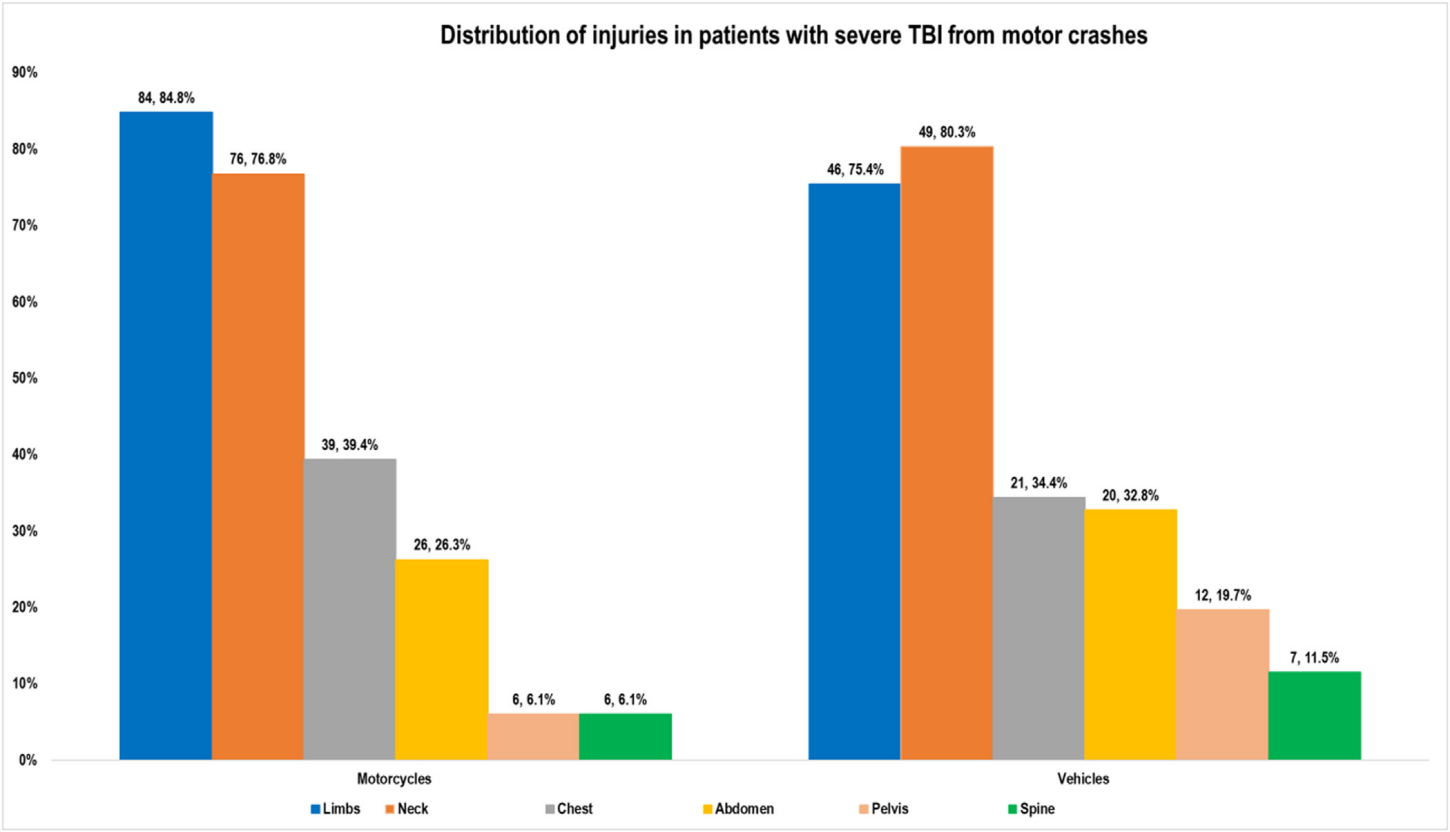
Bar graph comparing distribution of injuries in patients with severe traumatic brain injury from motorcycle and vehicle crashes.

The body regions that had the most associated injuries for motorcycle and vehicle crashes were the neck and limbs; and the least regions; the spine and pelvis. (Fig. 3)

At multivariable analysis, patients with severe TBI from vehicle crashes were 18% more likely to have polytrauma (adjusted PR = 1.18, 95% CI: 1.03-1.36, p-value = 0.018) when compared to those involved in motorcycle crashes (Table 3) when age was considered in the model.

**Table 3:** Multivariable analysis of the relationship of polytrauma in patients with severe traumatic brain injury to the mechanism of injury.

Characteristic	One injury (n=3)	More than one injury (n=157)	Adjusted PR	95% CI	p value
Age					
18-35	1 (1.0)	104 (99.0)	1	0.76-1.03	0.103
>35	2 (3.6)	53 (96.4)	0.88		
Mechanism					
Motorcycle	3 (3.0)	96 (97.0)	1		
Vehicle	0 (0.0)	61 (100.0)	1.18	1.03-1.36	0.018

Statistically significant if p-value < 0.05.

## Discussion

This study aimed at describing injury patterns in patients with severe traumatic brain injuries from motorcycle and motor vehicle RTCs admitted to Mulago Hospital Accidents & Emergency unit. Like similar studies on injury patterns from motorcycle RTCs in other settings, this study shows that victims of motorcycle RTCs with severe traumatic brain injuries mostly also sustain injuries to the extremities [19]. This reflects the population that has limited road safety measures and protective equipment. Extremities and torso region are vulnerable to injury in the unprotected settings of motorcyclists. In the context of overt severe traumatic brain injuries, attention can be focused on the management of the head in injuries. Some of the other more discrete injuries are unfortunately missed, with up to 8.8% missed injury rates recorded among multiply injured patients [20]. Identification of these injury patterns, in multiply injured patients with severe traumatic brain injuries, can facilitate more accurate triaging and definitive management of patients in A&E units because of the heightened sense of awareness of the likelihood of injuries.

The Berlin Polytrauma Definition describes polytrauma as injuries with an Abbreviated Injury Scale score of ≥3 in ≥2 body regions (2AIS ≥3) combined with the presence of ≥1 physiological risk factors [21]. Motorcyclists are among the most vulnerable groups of RTC victims. Given the inherent unsafety of this type of vehicle and its growing usage by the youths, there has been an increase in the incidence of RTCs involving these road users. The age of motorcyclist has been shown to affect the severity of injuries sustained. Young motorcyclists, with limited experience, have been reported to take more risks while riding, resulting in higher cases of catastrophic crashes. They are often prone to speeding and ignoring traffic and safety regulations. Other factors like non-helmet use increase the risk of the severe injuries [22].

### Demographic characteristics

#### Age

The results from this study indicated that most motorcycle RTCs involved the young; with a median age of 32 ([25]-39) years. The high probability of motorcycle crashes in younger individuals in Uganda could be attributed to their risky behaviours like overtaking, ignoring traffic rules and speeding. Majority of the active commercial motorcycle riders in Uganda are young men. Motorcycles are an affordable and popular source of earning especially in the urban areas, in comparison to motor vehicles. Previous studies have also shown that motorcycle road traffic crashes mostly happen among individuals of younger age group [23].

#### Sex

Majority of motorcycle RTC victims in this study were males, similar to the 80% male predominance reported in Kigali [24].This globally reported male predominance might be attributable to the fact that males are most commercial motorcycle riders in Uganda.

#### Use of protective gear

Wearing protective gear decreases the likelihood of sustaining several injuries among motorcyclists. Protective gear includes helmets, coveralls, overcoats, jackets, and heavy trousers. Only 19.2% of patients in this study wore helmets at the time of injury. Helmet use in the East Africa region is comparably higher (28%) in Western Kenya [25]. It is well established that helmets decrease the incidence and severity of traumas in motorcyclists. Those cyclists who do not wear such helmets are at a higher risk of sustaining severe head trauma, compared to the cyclists with helmets. The Traffic and Road Safety Act 1998 does not exclusively mandate the use of helmets for cyclists in Uganda [26]. Most of these patients wearing protective coveralls (57.1%) had a single limb injury or less. However, patients who were not wearing protective coveralls were more likely (10%) to have multiple limb injuries as compared to those who had protective coveralls.

#### Mode of transport

This study showed that only 36.9% of the patients were transported to the hospital by ambulance. This could be attributed to the low penetration of ambulances across the country, as the Ministry of Health emergency medical services system is still in its infancy. Majority of the patients were transported by unconventional modes of transport, by police pick-up trucks (40%).

#### Anatomical Sites of injuries

The limbs were the most injured body region (84.8%) in patients with severe traumatic brain injury resulting from motorcycle RTCs. These findings are consistent with a descriptive study on motorcycle crashes in Rwanda that reported a similar pattern of more injuries involving limbs [24]. The reason for the high vulnerability of motorcyclists to limb injuries may be that the limbs are exposed and therefore sites of impact during a crash. Soft tissue injuries, namely hematomas, abrasions, and lacerations, were documented in all regions; accounting for 67.4% of injuries in this study. A cross-sectional descriptive prospective study in Sri Lanka reported similar patterns of soft tissue injuries [19]. Initiatives should look at promoting efforts to minimise extremity and trunk injuries. Soft tissue injuries, if not appropriately identified and managed, predispose patients to infections and sepsis (46%) [27].Chest injuries, in this study, were slightly more ( (39.4%) in patients from motorcycle RTCs than in patients from vehicle RTCs (34.4%). The pelvis was one of the least injured regions (6.1%) regions of the body in this study among motorcyclists; like findings from a cross sectional study on 4,200 patients in Iran [28]. Equally few cases (6.1%) of spinal injuries were identified. It had been previously documented that protective helmets reduce the occurrence of cervical spine injuries [29].

#### Prevalence of polytrauma

In this study, patients with severe traumatic brain injury involved in vehicle RTCs were 18% more likely to have polytrauma when compared to those involved in motorcycle RTCS. Occurrence of polytrauma in motorcycle crashes has been documented in several studies. Given that a motor vehicle crash is high impact in an enclosed structure, it is plausible to have a higher risk of sustaining multiple injuries when compared to motorcycle crashes. A motorcycle crash might have fewer injuries because the impact of the crash throws the rider and passenger off the bike which limits the number of injuries, while in an enclosed compartment of a vehicle, the momentum of the crash is transmitted to the patient inside, hence more injuries. A retrospective review of motorcycle trauma at a tertiary care hospital in Pakistan reported that 19.5% of all victims had poly trauma [30]. Commonest regions affected are limbs, chest, and abdomen.

### Clinical application

The study identified predictors of multiple injuries in motorcycle crashes were age<35, low GCS, no helmet, or protective coveralls; and showed that victims of vehicle crashes have a higher likelihood of having polytrauma. The presence of these predictors may be utilised by clinicians in A&E units to thoroughly evaluate patients with severe head injuries for other injuries and provide appropriate care for all identified injuries.

### Strengths of the study

The study site was Mulago Hospital which receives patients referred from across the country. This ensures that the results can be generalisable to other parts of the country. The population was of patients with two mechanisms of injury (motorcycle and vehicle crashes) which provided for variety in clinical presentations. The assessment tool used was developed in accordance with physical examination standards in clinical practice to ensure that no injuries were missed. The findings in the patients’ charts were recorded following physical examination by doctors in the A&E units, followed by a repeat examination by the research assistants. All patients were still present in the hospital at the time of data extraction. Data were collected after a 24 hour period from admission into the Emergency Department to give time for the emergency unit team and other specialist firms to exam the patients and document their clinical findings. With this period, we increased the chances of capturing as much clinical findings as possible before the research assistant carried our examination.

### Limitations of the Study

This was a single-centre study, with a small sample size, although powered to meet the study objectives. Due to Mulago Hospital being a national referral hospital, there is a possibility of referral bias resulting in only patients with severe injuries being admitted to the hospital. The study also excluded data from motorcyclists who died on the scene of the crash or who were not admitted to the A&E . This means the results closely represent the clinical picture in other hospitals in the country. The skill of the research assistants might not be superior to the other clinicians, and we acknowledge that as a weakness to this study, but there are some injuries that are missed as clinicians focus on the head injury. Subsequent studies could improve the methodology by using more senior clinicians.

## Conclusion

This study showed that patients who sustain severe traumatic brain injuries from vehicle crashes have an increased likelihood of having multiple injuries, compared to patients from motorcycle RTCs. For motorcycle users, injuries most commonly affect the limbs. At particular risk are motorcyclists who do not wear helmets and protective coveralls.

## Recommendations

1 Medical teams working in A&E units should ensure thorough physical examination of patients with severe head injuries from motorcycle and motor vehicle crashes to identify accompanying injuries.
2 A road safety policy on the use of protective clothing for motorcyclists.
3 Further studies recommended with larger sample size.

## Dissemination of results

The result from this study were shared with Mulago Hospital and Ministry of Health Uganda.

## Authors’ contributions

Authors contributed as follow to the conception or design of the work; the acquisition, analysis, or interpretation of data for the work; and drafting the work or revising it critically for important intellectual content: JK contributed 60%, MN 10%, EO 10%, LW 10%, SM 5%, DO 5% each. All authors approved the version to be published and agreed to be accountable for all aspects of the work.

## Declaration of Competing Interests

Prof Wallis is an editor of the African Journal for Emergency Medicine. Prof Wallis did not participate in this manuscript's editorial process. The journal applies a double blinded process for all manuscript peer review. The authors declared no further conflicts of interest.
